# Supplementary material for: Generation and characterization of cross neutralizing human monoclonal antibody against 4 serotypes of dengue virus without enhancing activity
Source: PeerJ. 2017 Nov 13;5:e4021. doi: 10.7717/peerj.4021 (PMC5689018; doi:10.7717/peerj.4021)
Supplement: Table S1 [file peerj-05-4021-s001.docx]

**Supplementary Table 1**. Primers used for IgG isotype identification

| Primers | Sequences |
| --- | --- |
| Human IgG1&3 Fw | 5ʹ GTGACAAAACTCACACATG 3ʹ |
| Human IgG2 Fw | 5ʹ CAAATGTTGTGTCGAGTGC 3ʹ |
| Human IgG4 Fw | 5ʹ CAAATATGGTCCCCCATGC 3ʹ |
| Human IgG Rv | 5ʹ TTTGTCTTGGCATTATGCAC 3ʹ |

FW is forward primer, Rv is reverse primer

1
