# Supplementary figures and images for: Generation and characterization of cross neutralizing human monoclonal antibody against 4 serotypes of dengue virus without enhancing activity
Source: PeerJ. 2017 Nov 13;5:e4021. doi: 10.7717/peerj.4021 (PMC5689018; doi:10.7717/peerj.4021)

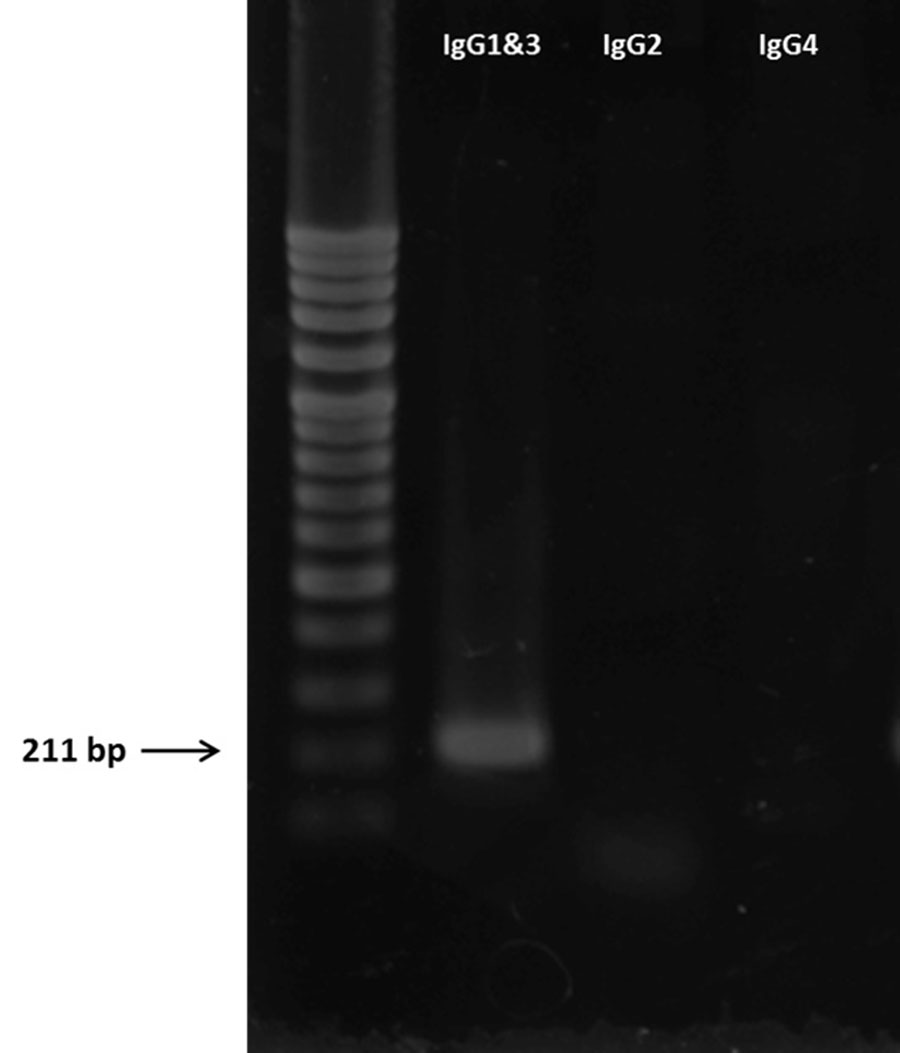

Supplement: Figure S1 — First lane was 100 bps DNA ladder. Second lane was the sample that amplified from the same forward primer of IgG1 and IgG3; third lane was the sample amplified by forward primer of IgG2 and last lane was the sample amplified by forward primer of IgG4. All of isotypes used the same reverse primer. The expected sizes of the PCR products were 211, 207, 346, and 210 bp for IgG1, IgG2, IgG3, and IgG4, respectively. [file peerj-05-4021-s002.jpg]
