# Supplementary material for: Generation and characterization of cross neutralizing human monoclonal antibody against 4 serotypes of dengue virus without enhancing activity
Source: PeerJ. 2017 Nov 13;5:e4021. doi: 10.7717/peerj.4021 (PMC5689018; doi:10.7717/peerj.4021)
Supplement: Supplemental Information 1 — The table shows foci number of triplicate obtained from 2 antibodies (B3B9 and N297Q-B3B9 rIgG) against DENV1. [file peerj-05-4021-s003.docx]

**Figure 2.** Neutralizing activity of B3B9 and N297Q-B3B9 rIgG antibody against four DENV serotypes.

The table shows foci number of triplicate obtained from 2 antibodies (B3B9 and N297Q-B3B9 rIgG) against DENV1

|  | DV1_B3B9 | | | DV1_N297Q-B3B9 | | |
| --- | --- | --- | --- | --- | --- | --- |
| antibody concentration (µg/ml) | FFU(1) | FFU(2) | FFU(3) | FFU(1) | FFU(2) | FFU(3) |
| 64 | 9 | 8 | 6 | 8 | 9 | 9 |
| 32 | 15 | 17 | 16 | 25 | 25 | 27 |
| 16 | 19 | 14 | 17 | 29 | 32 | 31 |
| 8 | 26 | 24 | 22 | 36 | 42 | 39 |
| 4 | 30 | 27 | 25 | 44 | 49 | 45 |
| 2 | 36 | 35 | 31 | 49 | 54 | 50 |
| 1 | 39 | 32 | 34 | 57 | 55 | 59 |
| 0.5 | 40 | 36 | 38 | 59 | 61 | 61 |
| 0.25 | 42 | 45 | 51 | 63 | 65 | 64 |
| 0.125 | 52 | 51 | 49 | 64 | 65 | 67 |
| 0.0625 | 55 | 57 | 61 | 63 | 67 | 66 |
| 0 | 57 | 60 | 62 | 69 | 71 | 73 |

The table shows foci number of triplicate obtained from 2 antibodies (B3B9 and N297Q-B3B9 rIgG) against DENV2

|  | DV2_B3B9 | | | DV2_N297Q-B3B9 | | |
| --- | --- | --- | --- | --- | --- | --- |
| antibody concentration (µg/ml) | FFU(1) | FFU(2) | FFU(3) | FFU(1) | FFU(2) | FFU(3) |
| 64 | 0 | 0 | 0 | 0 | 0 | 0 |
| 32 | 0 | 0 | 0 | 0 | 0 | 0 |
| 16 | 3 | 1 | 2 | 2 | 1 | 1 |
| 8 | 0 | 0 | 0 | 0 | 0 | 0 |
| 4 | 0 | 0 | 0 | 0 | 0 | 0 |
| 2 | 1 | 0 | 1 | 0 | 0 | 0 |
| 1 | 4 | 2 | 3 | 8 | 6 | 7 |
| 0.5 | 5 | 7 | 7 | 10 | 7 | 9 |
| 0.25 | 18 | 10 | 14 | 12 | 14 | 14 |
| 0.125 | 24 | 20 | 22 | 20 | 25 | 23 |
| 0.0625 | 29 | 26 | 27 | 24 | 26 | 27 |
| 0 | 52 | 33 | 39 | 52 | 31 | 42 |

The table shows foci number of triplicate obtained from 2 antibodies (B3B9 and N297Q-B3B9 rIgG) against DENV3

|  | DV2_B3B9 | | | DV3_N297Q-B3B9 | | |
| --- | --- | --- | --- | --- | --- | --- |
| antibody concentration (µg/ml) | FFU(1) | FFU(2) | FFU(3) | FFU(1) | FFU(2) | FFU(3) |
| 64 | 1 | 4 | 3 | 2 | 4 | 3 |
| 32 | 2 | 6 | 5 | 3 | 5 | 3 |
| 16 | 8 | 12 | 11 | 11 | 4 | 6 |
| 8 | 23 | 24 | 20 | 15 | 22 | 19 |
| 4 | 55 | 41 | 48 | 55 | 45 | 47 |
| 2 | 62 | 67 | 65 | 44 | 68 | 53 |
| 1 | 62 | 64 | 66 | 54 | 72 | 60 |
| 0.5 | 72 | 80 | 79 | 60 | 72 | 63 |
| 0.25 | 84 | 95 | 90 | 71 | 89 | 75 |
| 0.125 | 76 | 86 | 81 | 80 | 74 | 79 |
| 0.0625 | 85 | 92 | 87 | 88 | 82 | 80 |
| 0 | 120 | 114 | 111 | 113 | 117 | 109 |

The table shows foci number of triplicate obtained from 2 antibodies (B3B9 and N297Q-B3B9 rIgG) against DENV4

|  | DV4_B3B9 | | | DV4_N297Q-B3B9 | | |
| --- | --- | --- | --- | --- | --- | --- |
| antibody concentration (µg/ml) | FFU(1) | FFU(2) | FFU(3) | FFU(1) | FFU(2) | FFU(3) |
| 64 | 0 | 0 | 0 | 0 | 0 | 0 |
| 32 | 0 | 0 | 0 | 0 | 0 | 0 |
| 16 | 0 | 0 | 0 | 0 | 1 | 1 |
| 8 | 3 | 3 | 3 | 7 | 2 | 3 |
| 4 | 4 | 8 | 6 | 11 | 14 | 9 |
| 2 | 12 | 15 | 14 | 15 | 18 | 23 |
| 1 | 19 | 20 | 19 | 17 | 20 | 25 |
| 0.5 | 20 | 25 | 23 | 21 | 18 | 26 |
| 0.25 | 24 | 27 | 26 | 27 | 25 | 32 |
| 0.125 | 22 | 27 | 25 | 30 | 25 | 33 |
| 0.0625 | 33 | 32 | 32 | 32 | 33 | 37 |
| 0 | 37 | 28 | 33 | 33 | 34 | 43 |
