# Supplementary material for: Generation and characterization of cross neutralizing human monoclonal antibody against 4 serotypes of dengue virus without enhancing activity
Source: PeerJ. 2017 Nov 13;5:e4021. doi: 10.7717/peerj.4021 (PMC5689018; doi:10.7717/peerj.4021)
Supplement: Supplemental Information 2 — (A) ADE assay on K562 cells of B3B9 and N297Q_B3B9 rIgG against DENV1-4. (B) ADE assays in THP-1. [file peerj-05-4021-s004.docx]

**Figure 3**. ADE assay in K562 and THP-1 cells.

(A) ADE assay on K562 cells of B3B9 and N297Q_B3B9 rIgG against DENV1

|  | number of infected cells | | number of infected cells | |
| --- | --- | --- | --- | --- |
| Antibody concentration (µg/ml) | B3B9 rIgG(1)* | B3B9 rIgG (2)* | N297Q_B3B9 rIgG(1)* | N297Q_B3B9 rIgG (2)* |
| 400 | 75161 | 82238 | 7 | 3 |
| 100 | 86315 | 129165 | 11 | 5 |
| 25 | 173246 | 190171 | 2 | 1 |
| 6.25 | 257716 | 141474 | 9 | 1 |
| 1.56 | 113626 | 127550 | 10 | 5 |
| 0.39 | 112779 | 97086 | 15 | 4 |
| 0.097 | 89700 | 62083 | 11 | 11 |
| 0.024 | 18617 | 16155 | 22 | 32 |
| 0.006 | 2231 | 846 | 44 | 13 |
| 0.0015 | 58 | 97 | 7 | 1 |
| 0.00038 | 56 | 31 | 36 | 31 |
| 0 | 42 | 69 | 69 | 45 |

*The average number of infected cell counted from 3 frame at 20X magnification

ADE assay on K562 cells of B3B9 and N297Q_B3B9 rIgG against DENV2

|  | number of infected cells | | number of infected cells | |
| --- | --- | --- | --- | --- |
| Antibody concentration (µg/ml) | B3B9 rIgG(1)* | B3B9 rIgG (2)* | N297Q_B3B9 rIgG(1)* | N297Q_B3B9 rIgG (2)* |
| 400 | 3 | 1 | 1 | 1 |
| 100 | 5 | 1 | 1 | 1 |
| 25 | 1 | 1 | 1 | 1 |
| 6.25 | 1 | 5 | 1 | 1 |
| 1.56 | 34 | 95 | 1 | 2 |
| 0.39 | 9693 | 8539 | 23 | 26 |
| 0.097 | 26310 | 48004 | 769 | 769 |
| 0.024 | 9155 | 3923 | 1231 | 1769 |
| 0.006 | 1539 | 1846 | 692 | 308 |
| 0.0015 | 1231 | 1616 | 923 | 1077 |
| 0.00038 | 1231 | 1462 | 846 | 923 |
| 0 | 1154 | 1539 | 1616 | 1308 |

*The average number of infected cell counted from 3 frame at 20X magnification

ADE assay on K562 cells of B3B9 and N297Q_B3B9 rIgG against DENV3

|  | number of infected cells | | number of infected cells | |
| --- | --- | --- | --- | --- |
| Antibody concentration (µg/ml) | B3B9 rIgG (1)* | B3B9 rIgG (2)* | N297Q_B3B9 rIgG(1)* | N297Q_B3B9 rIgG (2)* |
| 400 | 1 | 4 | 1 | 1 |
| 100 | 24 | 31 | 1 | 2 |
| 25 | 385 | 615 | 1 | 1 |
| 6.25 | 4308 | 4077 | 1 | 1 |
| 1.56 | 8693 | 12078 | 1 | 1 |
| 0.39 | 38080 | 36465 | 1 | 1 |
| 0.097 | 32157 | 24310 | 15 | 7 |
| 0.024 | 2462 | 3385 | 19 | 13 |
| 0.006 | 462 | 615 | 29 | 2 |
| 0.0015 | 41 | 31 | 8 | 12 |
| 0.00038 | 39 | 43 | 15 | 26 |
| 0 | 55 | 64 | 39 | 34 |

*The average number of infected cell counted from 3 frame at 20X magnification

ADE assay on K562 cells of B3B9 and N297Q_B3B9 rIgG against DENV4

|  | number of infected cells | | number of infected cells | |
| --- | --- | --- | --- | --- |
| Antibody concentration (µg/ml) | B3B9 rIgG(1)* | B3B9 rIgG (2)* | N297Q_B3B9 rIgG(1)* | N297Q_B3B9 rIgG (2)* |
| 400 | 16463 | 12078 | 1 | 1 |
| 100 | 63852 | 66468 | 1 | 1 |
| 25 | 127550 | 99778 | 1 | 7 |
| 6.25 | 160091 | 16386 | 19 | 25 |
| 1.56 | 168784 | 183324 | 18 | 35 |
| 0.39 | 175323 | 173016 | 27 | 20 |
| 0.097 | 111779 | 138936 | 41 | 48 |
| 0.024 | 91701 | 87085 | 257 | 205 |
| 0.006 | 35311 | 46235 | 257 | 308 |
| 0.0015 | 6385 | 2769 | 400 | 51 |
| 0.00038 | 846 | 3231 | 308 | 400 |
| 0 | 462 | 923 | 462 | 512 |

*The average number of infected cell counted from 3 frame at 20X magnification

(B) ADE assays in THP-1

ADE assay on THP-1 cells of B3B9 rIgG antibody against DENV1. The viral RNA was quantified by a one-step Realtime PCR using glyceraldehyde 3-phosphate dehydrogenase (GADPH) as an internal control. Threshold cycle from duplicate experiment showed in the table was used for ΔΔCT analysis.

| Antibody  concentration(ug/ml) | CT of DENV(1) | CT of DENV(2) | Average | CT of GAPDH(1) | CT of GAPDH(2) | Average |
| --- | --- | --- | --- | --- | --- | --- |
| control | 29.71 | 29.98 | 29.845 | 18.81 | 18.73 | 18.77 |
| 100 | 19.71 | 20.04 | 19.875 | 22.12 | 21.92 | 22.02 |
| 10 | 18.42 | 19.09 | 18.755 | 19 | 19.3 | 19.15 |
| 1 | 20.62 | 20.73 | 20.675 | 19.11 | 19.43 | 19.27 |
| 0.1 | 29.54 | 29.78 | 29.66 | 19.39 | 19.46 | 19.425 |
| 0.01 | 28.51 | 31.56 | 30.035 | 19.25 | 19.64 | 19.445 |
| 0.001 | 29.5 | 30.48 | 29.99 | 19.11 | 19.05 | 19.08 |
| 0.0001 | 31.39 | 31.1 | 31.245 | 19.11 | 19.42 | 19.265 |

ADE assay on THP-1 cells of B3B9 rIgG antibody against DENV2. The viral RNA was quantified by a one-step Realtime PCR using glyceraldehyde 3-phosphate dehydrogenase (GADPH) as an internal control. Threshold cycle from duplicate experiment showed in the table was used for ΔΔCT analysis.

| Antibody  concentration (ug/ml) | CT of DENV (1) | CT of DENV (2) | Average | CT of GAPDH(1) | CT of GAPDH(2) | Average |
| --- | --- | --- | --- | --- | --- | --- |
| control | 26.53 | 26.41 | 26.47 | 18.65 | 18.83 | 18.74 |
| 100 | 31.04 | 30.68 | 30.86 | 18.35 | 18.43 | 18.39 |
| 10 | 25.16 | 25.18 | 25.17 | 18.41 | 18.73 | 18.57 |
| 1 | 20.63 | 20.56 | 20.595 | 18.3 | 18.26 | 18.28 |
| 0.1 | 24.02 | 24 | 24.01 | 18.25 | 18.52 | 18.385 |
| 0.01 | 25.98 | 25.57 | 25.775 | 17.96 | 18.37 | 18.165 |
| 0.001 | 25.41 | 25.09 | 25.25 | 18.05 | 18.19 | 18.12 |
| 0.0001 | 26.02 | 26.21 | 26.115 | 18.02 | 18.32 | 18.17 |

ADE assay on THP-1 cells of B3B9 rIgG antibody against DENV3. The viral RNA was quantified by a one-step Realtime PCR using glyceraldehyde 3-phosphate dehydrogenase (GADPH) as an internal control. Threshold cycle from duplicate experiment showed in the table was used for ΔΔCT analysis.

| Antibody  concentration(ug/ml) | CT of DENV (1) | CT of DENV (2) | Average | CT of GAPDH(1) | CT of GAPDH(2) | Average |
| --- | --- | --- | --- | --- | --- | --- |
| control | 33.19 | 34.15 | 33.67 | 18.81 | 19.21 | 19.01 |
| 100 | 25.49 | 25.61 | 25.55 | 20.46 | 20.88 | 20.67 |
| 10 | 21.58 | 21.9 | 21.74 | 18.64 | 18.68 | 18.66 |
| 1 | 26.22 | 26.74 | 26.48 | 18.4 | 18.5 | 18.45 |
| 0.1 | 32.54 | 33.51 | 33.025 | 18.39 | 18.63 | 18.51 |
| 0.01 | 43.61 | 34.41 | 39.01 | 18.25 | 18.48 | 18.365 |
| 0.001 | 33.7 | 34.33 | 34.015 | 18.39 | 18.53 | 18.46 |
| 0.0001 | 32.02 | 31.5 | 31.76 | 18.11 | 18.39 | 18.25 |

ADE assay on THP-1 cells of B3B9 rIgG antibody against DENV4. The viral RNA was quantified by a one-step Realtime PCR using glyceraldehyde 3-phosphate dehydrogenase (GADPH) as an internal control. Threshold cycle from duplicate experiment showed in the table was used for ΔΔCT analysis.

| Antibody  concentration(ug/ml) | CT of DENV (1) | CT of DENV (2) | Average | CT of GAPDH(1) | CT of GAPDH(2) | Average |
| --- | --- | --- | --- | --- | --- | --- |
| control | 20.15 | 20.14 | 20.145 | 17.27 | 17.3 | 17.285 |
| 100 | 10.96 | 11.5 | 11.23 | 19.3 | 19.4 | 19.35 |
| 10 | 11.26 | 11.29 | 11.275 | 17.77 | 18.01 | 17.89 |
| 1 | 13.07 | 12.95 | 13.01 | 16.9 | 17.08 | 16.99 |
| 0.1 | 15.85 | 16.07 | 15.96 | 17.3 | 17.19 | 17.245 |
| 0.01 | 20.11 | 20.28 | 20.195 | 17.31 | 17.35 | 17.33 |
| 0.001 | 20.01 | 20.09 | 20.05 | 16.99 | 16.97 | 16.98 |
| 0.0001 | 20.06 | 20.38 | 20.22 | 17 | 16.98 | 16.99 |

ADE assay on THP-1 cells of N297Q-B3B9 rIgG antibody against DENV1. The viral RNA was quantified by a one-step Realtime PCR using glyceraldehyde 3-phosphate dehydrogenase (GADPH) as an internal control. Threshold cycle from duplicate experiment showed in the table was used for ΔΔCT analysis.

| Antibody  concentation(ug/ml) | CT of DENV (1) | CT of DENV (2) | Average | CT of GAPDH(1) | CT of GAPDH(2) | Average |
| --- | --- | --- | --- | --- | --- | --- |
| control | 31.46 | 31.79 | 31.625 | 20.23 | 20.47 | 20.35 |
| 100 | 32.79 | 33.03 | 32.91 | 20.85 | 21.38 | 21.115 |
| 10 | 33.58 | 33.22 | 33.4 | 21.12 | 21.35 | 21.235 |
| 1 | 31.22 | 31.24 | 31.23 | 20.73 | 20.87 | 20.8 |
| 0.1 | 31.11 | 31.53 | 31.32 | 21.16 | 21.23 | 21.195 |
| 0.01 | 30.49 | 30.59 | 30.54 | 20.2 | 20.37 | 20.285 |
| 0.001 | 30.7 | 31.26 | 30.98 | 20.93 | 21.05 | 20.99 |
| 0.0001 | 30.53 | 31 | 30.765 | 20.87 | 20.66 | 20.765 |

ADE assay on THP-1 cells of N297Q-B3B9 rIgG antibody against DENV2. The viral RNA was quantified by a one-step Realtime PCR using glyceraldehyde 3-phosphate dehydrogenase (GADPH) as an internal control. Threshold cycle from duplicate experiment showed in the table was used for ΔΔCT analysis.

| Antibody  concentration(ug/ml) | CT of DENV (1) | CT of DENV (2) | Average | CT of GAPDH(1) | CT of GAPDH(2) | Average |
| --- | --- | --- | --- | --- | --- | --- |
| control | 27.11 | 27.43 | 27.27 | 19.32 | 19.27 | 19.295 |
| 100 | 29.71 | 30.35 | 30.03 | 20.31 | 19.92 | 20.115 |
| 10 | 29.03 | 29.3 | 29.165 | 18.93 | 20.11 | 19.52 |
| 1 | 26.69 | 26.93 | 26.81 | 18.88 | 19.04 | 18.96 |
| 0.1 | 26.68 | 26.78 | 26.73 | 18.64 | 19.43 | 19.035 |
| 0.01 | 24.99 | 24.89 | 24.94 | 18.54 | 18.55 | 18.545 |
| 0.001 | 27.29 | 27.1 | 27.195 | 19.17 | 19.01 | 19.09 |
| 0.0001 | 26.75 | 27.43 | 27.09 | 19.13 | 19.27 | 19.2 |

ADE assay on THP-1 cells of N297Q-B3B9 rIgG antibody against DENV3. The viral RNA was quantified by a one-step Realtime PCR using glyceraldehyde 3-phosphate dehydrogenase (GADPH) as an internal control. Threshold cycle from duplicate experiment showed in the table was used for ΔΔCT analysis.

| Antibody  concentration(ug/ml) | CT of DENV (1) | CT of DENV (2) | Average | CT of GAPDH(1) | CT of GAPDH(2) | Average |
| --- | --- | --- | --- | --- | --- | --- |
| control | 32.66 | 32.64 | 32.65 | 16.92 | 16.88 | 16.9 |
| 100 | 34.22 | 37.47 | 35.845 | 18.84 | 18.72 | 18.78 |
| 10 | 33.7 | 35.38 | 34.54 | 18.45 | 18.54 | 18.495 |
| 1 | 33.36 | 33.47 | 33.415 | 18 | 18.01 | 18.005 |
| 0.1 | 39.74 | 44.68 | 42.21 | 22.65 | 23.33 | 22.99 |
| 0.01 | 34.6 | 34.13 | 34.365 | 18.67 | 18.75 | 18.71 |
| 0.001 | 32 | 33.32 | 32.66 | 16.93 | 16.8 | 16.865 |
| 0.0001 | 32.58 | 31.96 | 32.27 | 16.84 | 16.9 | 16.87 |

ADE assay on THP-1 cells of N297Q-B3B9 rIgG antibody against DENV4. The viral RNA was quantified by a one-step Realtime PCR using glyceraldehyde 3-phosphate dehydrogenase (GADPH) as an internal control. Threshold cycle from duplicate experiment showed in the table was used for ΔΔCT analysis.

| Antibody concentration(ug/ml) | CT of DENV (1) | CT of DENV (2) | Average | CT of GAPDH(1) | CT of GAPDH(2) | Average |
| --- | --- | --- | --- | --- | --- | --- |
| control | 21.39 | 21.72 | 21.555 | 18.29 | 18.46 | 18.375 |
| 100 | 26.95 | 26.65 | 26.8 | 17.7 | 17.62 | 17.66 |
| 10 | 23.36 | 23.47 | 23.415 | 17.52 | 17.52 | 17.52 |
| 1 | 22.33 | 22.5 | 22.415 | 18.16 | 18.16 | 18.16 |
| 0.1 | 20.69 | 20.83 | 20.76 | 17.33 | 17.58 | 17.455 |
| 0.01 | 20.44 | 20.5 | 20.47 | 17.44 | 17.84 | 17.64 |
| 0.001 | 21.23 | 21.29 | 21.26 | 17.75 | 18.12 | 17.935 |
| 0.0001 | 20.61 | 20.75 | 20.68 | 17.65 | 17.89 | 17.77 |
