# Supplementary material for: Generation and characterization of cross neutralizing human monoclonal antibody against 4 serotypes of dengue virus without enhancing activity
Source: PeerJ. 2017 Nov 13;5:e4021. doi: 10.7717/peerj.4021 (PMC5689018; doi:10.7717/peerj.4021)
Supplement: Supplemental Information 3 [file peerj-05-4021-s005.docx]

**Figure 4D**. Raw data of phage inhibition ELISA. Different concentration of antibody were mixed with reciprocal concentration of 8 phage clones. Free phages were detected by ELISA. Each clone was performed in duplicate experiment.

| Antibody concentration (µg/ml) | 20 | 10 | 5 | 2.5 | 1.25 | 0.625 | 0.31 | 0.15 | 0.075 | 0.0325 | 0.016 | blank |
| --- | --- | --- | --- | --- | --- | --- | --- | --- | --- | --- | --- | --- |
| clone 1-1 | 0.596 | 0.671 | 0.754 | 0.876 | 0.945 | 1.069 | 1.031 | 1.102 | 1.177 | 1.181 | 1.134 | 1.173 |
| clone 1-2 | 0.572 | 0.655 | 0.744 | 0.842 | 0.936 | 1.019 | 1.097 | 1.087 | 1.137 | 1.121 | 1.131 | 1.153 |
| clone2-1 | 0.713 | 0.832 | 0.948 | 1.051 | 1.177 | 1.226 | 1.272 | 1.306 | 1.328 | 1.349 | 1.338 | 1.4 |
| clone2-2 | 0.7 | 0.825 | 0.941 | 1.053 | 1.253 | 1.216 | 1.277 | 1.296 | 1.327 | 1.346 | 1.347 | 1.366 |
| clone3-1 | 0.639 | 0.792 | 0.908 | 1.032 | 1.139 | 1.222 | 1.263 | 1.309 | 1.344 | 1.375 | 1.367 | 1.392 |
| clone3-2 | 0.648 | 0.797 | 0.925 | 1.036 | 1.141 | 1.21 | 1.269 | 1.314 | 1.339 | 1.364 | 1.363 | 1.397 |
| clone4-1 | 0.702 | 0.758 | 0.994 | 1.088 | 1.163 | 1.19 | 1.23 | 1.215 | 1.24 | 1.224 | 1.226 | 1.251 |
| clone4-2 | 0.647 | 0.828 | 1.004 | 1.086 | 1.176 | 1.21 | 1.222 | 1.231 | 1.238 | 1.226 | 1.21 | 1.225 |
| clone5-1 | 0.568 | 0.693 | 0.778 | 0.876 | 0.98 | 1.074 | 1.135 | 1.188 | 1.202 | 1.21 | 1.208 | 1.235 |
| clone5-2 | 0.559 | 0.681 | 0.762 | 0.854 | 0.964 | 1.046 | 1.13 | 1.159 | 1.184 | 1.21 | 1.22 | 1.237 |
| clone6-1 | 0.508 | 0.627 | 0.782 | 0.908 | 1.015 | 1.071 | 1.122 | 1.156 | 1.164 | 1.17 | 1.176 | 1.205 |
| clone6-2 | 0.512 | 0.639 | 0.788 | 0.925 | 1.028 | 1.101 | 1.147 | 1.161 | 1.171 | 1.182 | 1.177 | 1.194 |
| clone7-1 | 0.473 | 0.61 | 0.709 | 0.792 | 0.852 | 0.925 | 0.965 | 1.016 | 1.02 | 1.032 | 1.044 | 1.057 |
| clone7-2 | 0.484 | 0.612 | 0.702 | 0.783 | 0.864 | 0.943 | 0.973 | 1.018 | 1.046 | 1.04 | 1.049 | 1.109 |
| clone8-1 | 0.449 | 0.565 | 0.682 | 0.809 | 0.915 | 1.028 | 1.073 | 1.093 | 1.107 | 1.123 | 1.095 | 1.16 |
| clone8-2 | 0.451 | 0.558 | 0.672 | 0.813 | 0.929 | 1.019 | 1.059 | 1.086 | 1.114 | 1.118 | 1.099 | 1.099 |
| control | 1.163 | 1.383 | 1.3945 | 1.238 | 1.236 | 1.1995 | 1.083 | 1.1295 | 1.237 | 1.205 | 1.094 | 1.157 |
