# Supplementary material for: Generation and characterization of cross neutralizing human monoclonal antibody against 4 serotypes of dengue virus without enhancing activity
Source: PeerJ. 2017 Nov 13;5:e4021. doi: 10.7717/peerj.4021 (PMC5689018; doi:10.7717/peerj.4021)
Supplement: Supplemental Information 4 — The table shows foci number of triplicate of N297Q-B3B9 rIgG derived from a stable and transient CHO-K1 cell line against DENV2. [file peerj-05-4021-s006.docx]

**Figure 5.** The NT and ADE activity against DENV2 of N297Q-B3B9 rIgG derived from a stable and transient CHO-K1 cell line

The table shows foci number of triplicate of N297Q-B3B9 rIgG derived from a stable and transient CHO-K1 cell line against DENV2.

|  | N297Q_B3B9 rIgG from transient expression | | | N297Q_B3B9 rIgG from stable expression | | |
| --- | --- | --- | --- | --- | --- | --- |
| Antibody concentration (µg/ml) | FFU(1) | FFU(2) | FFU(3) | FFU(1) | FFU(2) | FFU(3) |
| 64 | 0 | 0 | 0 | 0 | 0 | 0 |
| 32 | 0 | 0 | 0 | 0 | 0 | 0 |
| 16 | 2 | 1 | 1 | 2 | 1 | 0 |
| 8 | 0 | 0 | 0 | 0 | 0 | 0 |
| 4 | 0 | 0 | 0 | 0 | 0 | 0 |
| 2 | 0 | 0 | 0 | 0 | 0 | 0 |
| 1 | 7 | 5 | 8 | 8 | 6 | 7 |
| 0.5 | 9 | 7 | 10 | 30 | 20 | 27 |
| 0.25 | 10 | 15 | 14 | 33 | 30 | 34 |
| 0.125 | 25 | 20 | 22 | 39 | 41 | 43 |
| 0.0625 | 27 | 21 | 27 | 53 | 51 | 57 |
| 0 | 33 | 39 | 52 | 50 | 31 | 44 |

ADE activity against DENV2 of N297Q-B3B9 rIgG derived from a stable and transient CHO-K1 cell line

|  | number of infected cells | | number of infected cells | |
| --- | --- | --- | --- | --- |
| Antibody concentration (µg/ml) | Stable expression(1)* | Stable expression(2)* | Transient expression(1)* | Transient expression(2)* |
| 400 | 1 | 1 | 1 | 1 |
| 100 | 1 | 1 | 1 | 1 |
| 25 | 1 | 1 | 1 | 1 |
| 6.25 | 1 | 1 | 1 | 1 |
| 1.56 | 1 | 2 | 1 | 2 |
| 0.39 | 23 | 26 | 23 | 26 |
| 0.097 | 769 | 769 | 769 | 769 |
| 0.024 | 1231 | 1769 | 1231 | 1769 |
| 0.006 | 692 | 308 | 692 | 308 |
| 0.0015 | 923 | 1077 | 923 | 1077 |
| 0.00038 | 846 | 923 | 846 | 923 |
| 0 | 1616 | 1308 | 1616 | 1308 |

*The average number of infected cell counted from 3 frames at 20X magnification.
